# Pain assessment and treatment in patients with mucopolysaccharidoses: a French multicentric pediatric study

**DOI:** 10.1186/s13023-025-04065-9

**Published:** 2025-11-12

**Authors:** Mélanie Blin, Marine Tardieu, Didier Lacombe, Magali Gorce, Léna Damaj, Magalie Barth, Delphine Genevaz, Sophie Vibet, François Labarthe

**Affiliations:** 1 Centre de Référence Maladies Héréditaires du Métabolisme ToTeM, CHRU Tours, Tours, France; 2 Inserm U1211, Université de Bordeaux, Centre de Compétences Maladies Héréditaires du Métabolisme, CHU de Bordeaux, Bordeaux, France; 3 Centre de Référence Maladies Héréditaires du Métabolisme, CHU Toulouse, Toulouse, France; 4 Centre de Compétences Maladies Héréditaires du Métabolisme, CHU Rennes, Rennes, France; 5 Centre de Compétences Maladies Héréditaires du Métabolisme, CHU Angers, Angers, France; 6 Vaincre les Maladies Lysosomales (VML), Massy, France; 7 Centre d’Évaluation et de Traitement de la Douleur, CHRU Tours, Tours, France; 8 N2C, Inserm U1069, Université François Rabelais de Tours, Tours, France

**Keywords:** Lysosomal storage disease, Children, Chronic pain, Practice assessment

## Abstract

**Background:**

Mucopolysaccharidoses (MPS) are a group of rare genetic lysosomal storage disorders with a wide spectrum of clinical severities. Chronic pain is frequent but difficult to assess. The aim of this study was to evaluate the detection and management of pain in pediatric MPS patients.

**Methods:**

Pain-related data were retrospectively collected from the medical records of pediatric MPS patients from five French centers for inborn metabolic disorders. A national online survey was also conducted about the feelings of patients and/or their families and of healthcare professionals about the detection and management of pain in pediatric MPS patients.

**Results:**

The medical records of 48 patients with all subtypes of MPS were analyzed. Pain was frequent and recurrent in MPS patients (pain was reported in 94% of the patients), but it was undoubtedly difficult to assess. We observed important differences between (1) medical records demonstrating frequent assessment and treatment of pain, (2) feelings of patients or their families (53 questionnaires) reporting frequent pain, and (3) feelings of healthcare professionals (21 questionnaires) who were quite satisfied with their own practices, suggesting that the majority of patients were unpainful. We recommend a more systematic evaluation of pain, particularly for outpatients, with the use of adapted tools, notably in children with disabilities, and with a multidimensional approach to pain assessment and management. Caregiver training is also needed, and close collaboration with pain centers is encouraged.

**Conclusion:**

A routine pain assessment protocol for MPS patients is required that covers the entire spectrum of pain and can be adapted for every type of patient, including those with neurocognitive and motor impairments.

**Supplementary information:**

The online version contains supplementary material available at 10.1186/s13023-025-04065-9.

## Highlights

- Chronic pain is a prevalent feature in pediatric MPS patients, but its detection and management remain complex.
- Specific tools are required for the detection of pain in these patients; these tools may be adapted for patients with neurocognitive impairment and may constitute a multidimensional approach to pain assessment and management (complex model considering nociceptive, affective, sociocultural, cognitive and behavioral approaches).
- Caregiver training is needed, and close collaboration with pain centers is encouraged.

**Supplementary information:**

The online version contains supplementary material available at 10.1186/s13023-025-04065-9.

## Introduction

Mucopolysaccharidoses (MPS) are a group of rare genetic lysosomal storage disorders. The seven types of MPS exhibit a wide spectrum of clinical severity, including cognitive impairment, skeletal and joint abnormalities, short stature, coarsened facial features, vision loss, and cardiovascular and respiratory impairment [1, 2]. Chronic pain, defined as pain lasting more than 3 months) is a prevalent feature in MPS children, with various types of pain, including nociceptive pain (such as joint stiffness or visceral pain caused by organomegaly), neuropathic pain (e.g., carpal tunnel syndrome or spinal cord injuries), and sensory hypersensitivity [3, 4]. However, detecting and managing chronic pain in these patients may be difficult, especially in those with cognitive impairment [5–8]. Nevertheless, better pain management improves quality of life, as demonstrated in a subpopulation of MPS IV-A patients [9].

While many studies have suggested that MPS patients often experience chronic pain, few have assessed its detection and management [3, 6–10]. The present study aimed to retrospectively collect pain-related data (evaluation and treatment) from the medical records of patients at five French centers specializing in pediatric MPS management. The study also aimed to assess patients’ and/or their families’ and healthcare professionals’ feelings about the detection and management of pain during the follow-up of pediatric MPS patients using a national online survey.

## Patients and methods

### Retrospective analysis of medical records

We retrospectively collected pain-related data from the medical records of pediatric MPS patients recruited from five centers for inborn metabolic disorders (Angers, Bordeaux, Rennes, Toulouse, and Tours). The inclusion criteria were all types of MPS confirmed by genotype or metabolic profile, aged less than 19 years at inclusion and with more than 2 follow-up visits in the center. Patients or their parents who refused to participate were not included. Informed consent was obtained from each patient and their parents. The study was approved by the local Ethics Committee in Human Research of the University Hospital of Tours (Ref. RNIPH21-AIE D MPS).

Data were collected from the medical records of each patient during the last 5 years before inclusion. Specifically, we selected the 3 most recent visits of each type (i.e., outpatient consultation, day care clinic visits and inpatient hospitalization). In addition to patient characteristics, we collected pain-related data at each visit. Namely, pain assessment was defined by the use of a pain assessment scale during the visit or the presence of a pain-related word (“douleur (pain)”, “douloureux (painful)”, “indolore (painless)”, “algique (algic)”, “antalgique (analgesic)”, “analgésie (analgesia)”, or “paresthésie (paresthesia)”) in the medical records. The prescription of an antalgic treatment (in the presence of pain) and the eventual reassessment of pain after treatment were also collected.

### Online survey for patients and healthcare professionals

To better understand the feelings of patients or their parents and of healthcare professionals about their practices in the management of pain, we also conducted two different online surveys using a website platform (https://www.survio.com) [11]. All participation was anonymous and free of charge. An information letter and a link to join the online questionnaire were distributed via a mailing list from all the French centers for inborn errors of metabolism (Filière G2M, https://www.filiere-g2m.fr) and via the newsletter and an e-mail campaign aimed at members of the association of patients “Vaincre les maladies lysosomales” (https://www.vml-asso.org) [12, 13]. A recall was made 15 days after the first distribution of the survey.

The questionnaire for parents and patients (available in the supplementary material section of the journal) consists of 26 multiple-choice or free-choice items concerning the medical history of the patients and their feelings about pain management, from pain expression, the method used to detect and estimate the frequency, to the eventual proposed treatments and their efficacy. The questionnaire for healthcare professionals (available in the supplementary material) was composed of 19 multiple-choice or free-choice items regarding practices and feelings about the management of pain in MPS patients.

### Statistical analysis

The results are expressed as the median [min–max] or number of subjects (%) as appropriate. Quantitative data were compared using a Kruskal‒Wallis test, with Dunn’s multiple comparison test when the first analysis indicated a significant difference. Qualitative data were compared using a chi-square test. The Spearman test was used for correlation analysis. All the statistical analyses were carried out using GraphPad Prism version 6.0 (GraphPad Software, Inc.). A *p* value < 0.05 was considered to indicate statistical significance.

## Results

### Retrospective analysis of pain assessment and treatment (from medical records)

#### Patient characteristics

Forty-eight MPS patients were enrolled in this retrospective study. Patient characteristics are presented in Table 1. Briefly, MPS-I was the most common disease, followed by MPS-II and MPS-III, whereas MPS-IV, MPS-VI and MPS-VII were the most marginal. There was a predominance of males, probably because of the X-linked transmission of MPS-II (9 of the 10 MPS-II patients were males). Twenty-two patients (46%) were currently treated with enzyme replacement therapy (ERT, 8 MPS-I, 8 MPS-II, 3 MPS-IV, and 3 MPS-VI), and 13 (27%) were treated with hematopoietic stem cell transplantation (10 MPS-I and 2 MPS-II).

**Table 1:** Characteristics of the patients (pain-related data from medical records)

Characteristics	n (%)
Number of patients	48
Gender male/female	32 (67%)/16 (33%)
Age at diagnosis (months)	36 [0;189]
Age at inclusion (months)	119 [14;223]
Duration of follow-up (months)	68 [2;1459]
MPS type	
type I	19 (40%)
type II	10 (21%)
type III	11 (23%)
type IV	4 (8%)
type VI	3 (6%)
type VII	1 (2%)
Enzyme replacement therapy (ERT)	22 (46%)
Hematopoietic stem cell transplantation (HSCT)	13 (27%)

The results are expressed as the number of patients (% of responders) or median [min;max]

Each patient exhibited a wide spectrum of clinical severity (Table S1, supplementary material), including skeletal and joint abnormalities, ENT manifestations, cardiovascular and respiratory symptoms, and cognitive impairment. Thirty-eight percent of patients were diagnosed with carpal tunnel syndrome. Most of the patients required specific equipment (including wheelchairs, orthopedic aids, noninvasive ventilation, hearing aids, totally implantable venous access devices, etc.) and were managed with supportive care (Table S2 supplementary material). Fourteen patients (29%) regularly used analgesic treatments, mostly acetaminophen/paracetamol and NSAIDs, whereas opioid agents were not used. Among antineuropathic treatments, gabapentin and amitriptyline were exceptionally used, and one patient was treated with cannabidiol. Five patients (10%) had already received a specialized pain consultation before inclusion.

#### Pain assessment and treatment

**Fig. 1:**
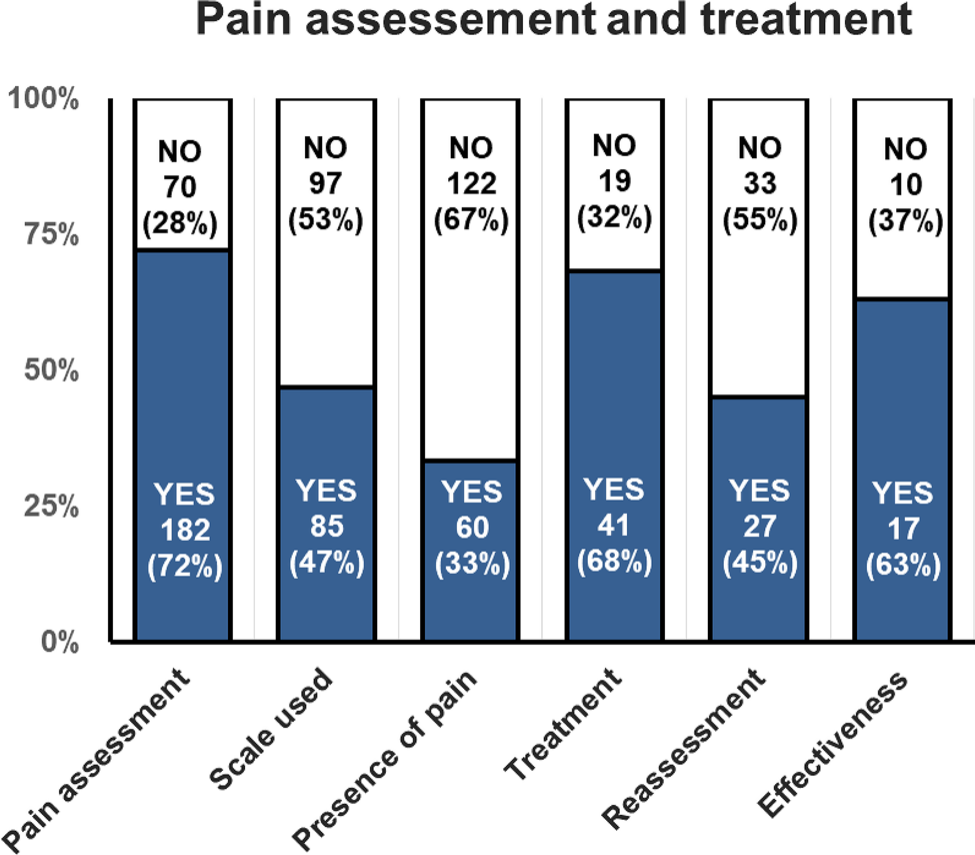
Pain assessment and treatment in MPS patients during visits at the reference center for inborn errors of metabolism. Data were extracted from the medical records of 252 visits at the reference centers for 48 MPS patients. The results are expressed as the number of visits (percentages calculated on the total of concerned visits)

We analyzed 252 visits at the reference centers for the 48 MPS patients, including 89 outpatient consultations, 98 day care hospitalizations and 65 inpatient hospitalizations. The results of the global pain assessment and treatment are presented in Fig. 1. Pain assessment was specified in 182 visits (72%), and the method used for assessment was detailed in only half of the cases. The main scale used to assess pain intensity was the Face Pain Scale Revisited (FPS-R, 34%), followed by the FLACC (24%), EVENDOL (15%), Numerical Rating Scale (13%) and Simple Verbal Scale (8%). More rarely, the HEDEN, EDIN, OPS and CHEOPS scales were used. When evaluation was done, pain was reported in 60 cases (33%), corresponding to 45 patients (94% of all patients). An analgesic treatment was proposed for two-thirds of the patients with a pain score > 0. Of the 41 therapeutic actions carried out in the presence of pain, the prescription of an analgesic drug was performed in 33 cases, whereas 3 patients were referred for specialized pain consultation, and physiotherapy sessions were proposed in 5 patients. Of the patients who were prescribed treatment, pain reassessment was documented in the medical record in only 66% of cases. Of those who underwent reassessment, treatment was deemed effective in 17 cases (41%).

The results considering the type of hospital visit are presented in Table 2. Day care hospitalization was the most frequent type of visit, whereas the number of inpatient hospitalizations was lower. Pain assessment was performed in the majority of day care and inpatient hospitalizations but was significantly less frequent during outpatient consultations. The method used for pain assessment was specified in most of the cases during inpatient hospitalizations but most rarely during day care hospitalizations or outpatient consultations. Finally, pain was more common during outpatient consultation than during inpatient hospitalization or day care hospitalization. The percentage of therapeutic interventions, reassessment and therapeutic efficacy were not significantly different among the 3 types of visits.

**Table 2:** Pain assessment and treatment

	Outpatient consultation	Day care hospitalization	Inpatient hospitalization	p
Number. of patients	39 (81%)	42 (88%)	35 (73%)	0.19
Number of visits	89 (35%)*	98 (39%)	65 (26%)**	< 0.01
Pain assessment	43 (48%)***^###^	82 (84%)	57 (88%)	< 0.0001
Scale used specified	13 (30%)^###^	32 (39%)^###^	40 (70%)	< 0.0001
Presence of pain	24 (56%)***	19 (23%)	17 (30%)	< 0.01
Therapeutic intervention	18 (75%)	8 (42%)^###^	15 (88%)***	< 0.01
Reassessment	11 (61%)	4 (50%)	12 (80%)	0.30
Effectiveness	6 (55%)	2 (50%)	9 (75%)	0.50

The results are expressed as numbers (%), with percentages related to the total number of patients or visits (lines 1 to 3), the number of pain assessments (lines 4 and 5) or the number of events from the previous line (lines 6 to 8). Therapeutic interventions included drug and nondrug interventions. The data were compared using a chi-square test (p), with a post hoc test when the first comparison was significant. *p < 0.05, ** *p* < 0,01, ***p < 0,001 versus day care hospitalization, ^###^*p* < 0,001 versus inpatient hospitalization

#### Factors related to pain assessment and treatment

We therefore analyzed the factors (other than the type of visit) that potentially modulate pain assessment and treatment. No significant difference was observed between the centers. Pain assessment was less frequently performed in MPS-III patients (59% of all visits) than in MPS-I, MPS-II and MPS-IV patients (72, 79 and 88%, respectively, *p* < 0.01). This lower frequency of pain assessment for MPS-III patients was observed regardless of the type of visit. However, when pain assessment was performed, no difference existed in the percentage of painful patients between the various types of MPS. Other factors are presented in Table 3. Briefly, pain assessment was more frequently performed for disabled patients requiring specific equipment, for patients treated by ERT (probably related to recurrent visits in day care clinics), or for those with cardio-respiratory symptoms or carpal tunnel syndrome. In contrast, pain assessment was less frequently performed in patients with delayed psychomotor development. The presence of pain was logically more frequent in patients with a history of chronic analgesic treatment or previous consultation in a pain center, skeletal and joint abnormalities or carpal tunnel syndrome and in older patients or those with a longer duration of follow-up. In contrast, the presence of pain was negatively correlated with psychomotor delay. Multivariate analyses were also performed but did not improve the results with only one significant factor in each multivariate model.

**Table 3:** Univariate analysis of factors correlated with pain assessment and presence of pain

Correlated variables	Spearman correlation coefficient (rho)	*p*-value
**Correlated with pain assessment**		
Equipment*	0.379	0.005
Enzyme Replacement Therapy (ERT)	0.379	0.008
Respiratory symptoms	0.362	0.012
Cardiologic symptoms	0.357	0.013
Carpal Tunnel Syndrome (CTS)	0.354	0.014
Delayed psychomotor development	−0.291	0.045
**Correlated with presence of pain**		
Previous consultation in a pain center	0.388	0.008
Carpal Tunnel Syndrome (CTS)	0.378	0.010
Delayed psychomotor development	−0.368	0.013
Chronic analgesic treatment	0.358	0.016
Age	0.346	0.020
Orthopedic symptoms	0.309	0.039
Duration of follow up	0.296	0.049

Univariate analyses were performed using a Spearman test (rank-order correlation). Only significant results are presented. *Equipment includes wheelchairs, hearing aids, glasses, implantable chambers, orthopedic aids, and noninvasive ventilation

### National survey (online questionnaire) for patients/families and healthcare professionals

We first evaluated the feelings of the patients and/or their families about pain assessment and treatment by the medical team. The results of this questionnaire are presented in Table 4. Fifty-three questionnaires were completed, most of which were by parents, and were representative of several MPS types. Patients had various ways to express pain, especially by oral expression or grimacing. Pain was frequently reported with a high intensity. Pain was variously considered by medical teams. The pain assessment was mostly performed by discussion with the patient and/or his family, and pain scales were rarely used. In the presence of pain, a treatment was proposed by the medical team, mostly involving the use of either nonopioid analgesics or sometimes nondrug therapies. Weak and strong opioid agents were proposed for 19 and 13% of the patients, respectively. One-third of the patients recognized automedication.

**Table 4:** Results of the online questionnaire for patients or their families

Characteristics of responders					
	Parents	Patient	Other		
*Responders*	42 (79%)	10 (19%)	1 (2%)		
	MPS-I	MPS-II	MPS-III	MPS-IV	MPS-VI
*Type of MPS*	23 (43%)	15 (28%)	11 (21%)	3 (6%)	1 (2%)
	Male	Female			
*Gender of patient*	35 (66%)	18 (33%)			
**Pain description**					
**Patient expression of pain (multiple answers possible)**					
*By words/phrases (e.g.: “I’m in pain” etc.)*	28 (53%)				
*By the face, grimaces, tensing of the face*	22 (42%)				
*By shouting, moaning, or crying*	15 (28%)				
*By a change of mood*	15 (28%)				
*By gestures, spasms, agitation*	11 (21%)				
*Other*	3 (6%)				
*Don’t know*	2 (4%)				
**Presence of pain in the last 2 years**	46 (87%)				
*≥ once a month*	37 (70%)				
* < once a month*	16 (30%)				
**Estimated pain intensity**	0–2/10	3–5/10	≥6/10		
*By Numerical Rating Scale (0 to 10)*	3 (6%)	11 (21%)	39 (74%)		
**Pain assessment**					
	Never	Rarely	Often	Always	Unknown
*Consideration of pain by the medical team*	2 (4%)	7 (13%)	24 (45%)	14 (26%)	6 (11%)
*Pain assessment in consultation/hospitalization*	6 (11%)	14 (26%)	16 (30%)	13 (25%)	4 (8%)
**Pain assessment approach**					
*By discussion between the patient and the medical team*	43 (81%)				
*By using a specific tool (pain scale)*	9 (17%)				
**Pain management**					
	Never	Rarely	Often	Always	
*Drug prescription by medical team in case of pain*	2 (4%)	11 (21%)	19 (36%)	21 (40%)	
*Previously referred to a pain center*	10 (19%)				
**Pain medication provided in the last 2 years**					
Nonopioid analgesics (class I)					
*Paracetamol/acetaminophen*	46 (87%)				
*Nonsteroidal anti-inflammatory drugs (ibuprofen, ketoprofen, niflumic acid, diclofenac)*	22 (42%)				
*Weak opioid agents (class II analgesics: tramadol, codeine)*	10 (19%)				
*Strong opioid agents (class III analgesics: morphine)*	7 (13%)				
Other drugs (gabapentin, amitriptyline, phloroglucinol, other*)	15 (28%)				
Nondrug treatment of pain**	38 (72%)				
**Frequency of use of nondrug analgesics**					
*≥ once a week*	27 (53%)				
* < once a month*	8 (15%)				
**Self-medication**					
*Drug analgesic*	15 (28%)				
*Nondrug analgesic*	20 (38%)				

The results are expressed as the number of patients (% of responders). *Other drugs include homeopathy, nitrous oxide and cannabidiol oil; **Nondrug treatments include physiotherapy, osteopathy, homeopathy, music therapy, hypnosis and/or sophrology

The results from the questionnaire for healthcare professionals are presented in Table 5. Twenty-one questionnaires were completed by 12 physicians and 9 nurses, most of whom had real experience in the follow-up of MPS patients. Most of the responders seemed to be comfortable with pain management for MPS patients because of the availability of local helpful tools, such as protocols, dedicated consultations, or training. Seventeen of them (81%) estimated that pain assessment was regularly performed (“sometimes” or “often”), and 16 healthcare professionals (76%) estimated that less than 50% of patients experienced pain. They were also used to assess pain and to treat it, either with drug or non-drug treatments. Finally, a majority of professionals (approximately two-thirds) were satisfied with their practice related to pain management.

**Table 5:** Results of the online questionnaire for healthcare professionals

Characteristics of responders					
	Physician	Nurse			
*Responders*	12 (57%)	9 (43%)			
	< 5 patients	5–10 patients	> 10 patients		
*Experience with MPS (number of patients)*	2 (10%)	8 (38%)	11 (52%)		
**Local helpful tools for pain management**					
*Current pain management protocols*	13 (62%)				
*Specialized pain consultation*	17 (83%)				
*Specific formation obtained*	10 (48%)				
**Pain assessment**					
	0–25%	25–50%	50–75%	> 75%	
*Estimated proportion of MPS patients with pain*	8 (38%)	8 (38%)	4 (19%)	1 (5%)	
**Pain assessment**	Never	Rarely	Sometimes	Often	Always
*Assessment during follow-up (global evaluation)*	1 (5%)	2 (10%)	12 (57%)	5 (24%)	1 (5%)
*Assessment at least annual*	3 (14%)	10 (48%)	8 (38%)	0 (0)	0 (0)
*Assessment because requested by the patient/parents*	1 (6%)	1 (6%)	0 (0)	2 (13%)	12 (75%)
*Assessment because of clinical symptoms*	2 (12%)	0 (0)	2 (12%)	4 (24%)	9 (53%)
*Assessment systematic at each visit*	2 (12%)	5 (29%)	6 (35%)	2 (12%)	2 (12%)
	*Visual Analog Scale*	*Face scale*	*FLACC*	*San Salvadour*	*GEDI, other*
*Which type of pain scale is used?*	13 (62%)	11 (52%)	5 (24%)	4 (19%)	0 (0)
**Pain treatments familiar to the prescriber**					
**Drugs of pain**					
*Nonopioid analgesics (class I analgesics)*	21(100%)				
*Weak opioid agents (class II analgesics)*	21(100%)				
*Strong opioid agents (class III analgesics)*	14 (67%)				
*Other drugs (including antineuropathic drugs and nitrous oxide gas)*	13 (62%)				
**Other treatments of pain**					
*Hypnosis*	11 (52%)				
*Physiotherapy*	7 (33%)				
*Nitrous oxide*	3 (14%)				
*Virtual reality headset*	3 (14%)				
*Relaxation techniques*	2 (10%)				
*Transcutaneous Electrical Nerve Stimulation*	1 (8%)				
**Self-evaluation of your practice** *(0 is very bad, 10 is very good)*?					
	0–4	5–10			
*Pain assessment (0 to 10)*	7 (35%)	13 (65%)			
*Pain management (0 to 10)*	6 (32%)	13 (68%)			

The results are expressed as the number of patients (% of responders)

## Discussion

Our study demonstrated that pain was a recurring issue among pediatric MPS patients. Although studies focusing on pain assessment in MPS patients are scarce, the existing literature consistently reports frequent pain among these patients [3, 6–10]. Our analysis of medical records revealed that pain management, including detection, evaluation and treatment, was routinely implemented without significant differences among MPS subtypes (except for less frequent pain assessments for MPS-III patients) or health centers. What makes our study original is that our analysis of medical records revealed significant discrepancies between (1) medical records demonstrating frequent pain assessment and treatment, (2) patient or family reports of frequent pain, and (3) healthcare professional perception that less than half of patients reported pain and satisfaction with their practices. This discrepancy raises the question of how we can improve our pain management practices for this patient group. To address this issue, we offer several suggestions.

First, pain assessment was performed in most day care and inpatient hospitalizations, but significantly less frequently during outpatient consultations. This may be because assessment of pain has been part of the quality and safety indicators used by the French “Haute Autorité de Santé” (HAS) to evaluate the quality of care in healthcare facilities for many years [14]. However, the HAS does not require this indicator for outpatient consultations. For this reason, pain assessment is probably performed less frequently. Another explanation could be that outpatient consultations have fewer healthcare professionals available, and medical teams focus on what they consider more urgent issues [7]. Raising healthcare professionals’ awareness of the need to assess pain during outpatient consultations will be our first recommendation.

Another point of discussion is the method used to assess pain. Our analysis of medical records revealed that pain was assessed less frequently in MPS-III patients. However previous studies have reported that pain is a common problem in these patients, particularly in older patients with severe developmental delay [3, 6, 7, 15, 16]. This discrepancy may be due to the overrepresentation of outpatient consultations compared to day care and inpatient admissions in MPS-III patients. This is likely due to the absence of specific treatment that require recurrent hospital admissions, such as ERT for other MPS subtypes. However, the neurocognitive deficits common in these patients make evaluating pain challenging and may contribute to pain being evaluating less frequently in MPS-III patients [6, 7, 17]. Pain is difficult to identify and treat appropriately in children with cognitive and communication impairments [6, 7, 17]. Specific scales and caregiver training are required to evaluate pain in children with disabilities [3, 18–20]. Our questionnaire results showed that the majority of caregivers were unaware of specific tools for pain assessment in children with disabilities, indicating the need to educate healthcare professionals on this topic. Furthermore, fewer than half of the cases specified the scales used for pain assessment, with no possibility of verifying that the scale was appropriate. Inadequate tools lead to inaccurate pain assessment and treatment for children with intellectual disabilities [19]. We recommend specifying the type of scale used, with a focus on children with cognitive impairment, who require specific scales.

Another explanation for underestimating chronic pain should be to consider only the sensori-discriminative part of pain and not the psycho-emotional, cognitive and behavioral dimensions of pain. Assessing the sensory-discriminative part of pain also requires multiple evaluations for i) the type of pain (nociceptive or neuropathic pain with the pediatric DN4 scale), ii) pain intensity (Faces Pain Scale, visual analog scale, numeric pain rating scale, … ), and iii) pain localization (body areas). Last but not least, pain evaluation also requires a quality of life scale, which assesses the effects of pain on social life, sleep, mood and mental health. These evaluations should also include the behavioral and social dimensions of pain. The previously cited scales have a good clinical correlation in children, although higher ratings were reported with the Numerical Pain Rating Scale [21]. Most participants in our online questionnaire for patients and families reported a high intensity of pain. These scores may seem to be high. However, the pain score was reported to be at this level in children experiencing chronic pain regardless of the etiology, as demonstrated in a pediatric cohort of 2249 children where the mean pain score was 6.4/10 [22]. The discrepancy between patients’ and/or their families’ feelings and the lower perception of pain among professionals underscore the need for a more comprehensive, multidimensional approach to pain assessment and management. Additionally, lysosomes play an important role in sensory neurons, especially in dorsal root ganglion, and lysosomal dysfunction has been suspected to contribute to chronic pain in general [23]. Pain in MPS patients extends beyond physical aspects, involving behavioral, social, and psychological dimensions [24]. The Pediatric Pain Profile (PPP) is suggested as a suitable tool for assessing acute or chronic pain in MPS patients, encompassing the multifaceted nature of pain [25, 26]. This scale is designed for the usual caregiver (most often the parent) and is used to assess pain in children with cognitive and motor disabilities, with a prior assessment carried out in a nonalgesic phase. It could be proposed to evaluate acute or chronic pain in hospitals or at home. Some vigilance indicators of pain in MPS patients could include orthopedic injuries, carpal tunnel syndrome, and/or equipment use. Our clinical goal is to maintain a two-dimensional approach to pain, both sensori-discriminative and psychological, which fits into a more complex model considering the sensori-discriminative, affective, sociocultural, cognitive and behavioral approach [27].

Nevertheless, our results also highlight the need to improve communication with patients and/or their families regarding pain detection and management, considering that healthcare professionals underestimate feelings of pain in MPS patients. We recommend addressing this topic during each consultation. We also suggest raising healthcare professionals’ awareness of the high frequency of pain in MPS patients and of the difficulty of its assessment [6, 7].

Drug therapies used in our population were mainly represented by nonopioid analgesics (class I according to the WHO classification), with frequent automedication recognized by patients or their families. While practitioners were aware of other analgesic therapies, they did not rate themselves as comfortable with prescribing them. Only one patient received cannabinoid oil for neuropathic pain, although experience with this treatment remains very limited in pediatric patients [28]. In addition to conventional MPS treatments, such as ERT, which has demonstrated beneficial effects on pain prevention [29, 30], new therapeutic options for pain control are in progress. High TNF-α levels have been linked to pain in MPS patients, reflecting chronic inflammation. The administration of anti-TNF-α treatment was associated with a decrease in joint pain and an improvement in mobility in animal models [31]. To our knowledge, this type of therapy is not currently used in human MPS patients. Furthermore, the use of nondrug therapies, such as chiropractics and massage, has remained very limited, whereas their use has been associated with a reduction in pain intensity and anxiety in children [32–34]. The cost of these nondrug therapies remains high for patients.

Finally, referrals to pain centers remain limited, possibly related to access difficulties according to comments in our patient/family questionnaire, even if a large majority of healthcare professionals declared having access to a specialized pain consultation and half of them had undergone a specific pain formation. The new French guidelines for chronic pain management recommend involving the general practitioner in the first instance, followed by a pain consultation if necessary. If this proves insufficient, a third-level evaluation at a pain management center is recommended [35]. As we can see in this study, MPS can lead to chronic pain, and collaboration among pediatricians, general practitioners and doctors in pain management centers is needed to improve access to nondrug therapies when cost is a problem.

We recognize that our study has several limitations. First, patients and/or their families who completed the online questionnaire were not strictly the same as patients for whom medical records were studied. In fact, medical records were obtained from five centers for inborn errors of metabolism, whereas the online questionnaire was anonymous and distributed nationally via the French network for inborn errors of metabolism “Filière G2M” and via the association of patients “Vaincre les maladies lysosomales”. Approximately fifty patients were enrolled in each part of the study, which was a small sample but also varied enough (considering all the MPS subtypes and from at least five different centers) to be representative of French MPS patients. The retrospective methodology of our study was also another limitation, and we cannot exclude the possibility that patients were asked for pain during medical visits even if there was no mention of pain in their medical records. Finally, a quality of life questionnaire was not routinely used during the follow-up of these patients, although we know that the persistence of chronic pain may alter their quality of life even if some symptoms are improved by specific therapies such as ERT or hematopoietic stem cell transplantation [36]. However, all recent guidelines recommend performing repeated quality of life evaluations during the follow-up of MPS patients [2, 37].

## Conclusion

In summary, our study demonstrated that the feeling of pain was frequent and recurrent in MPS patients but was undoubtedly difficult to assess. Indeed, pain detection and treatment were defective in our current practice, with a gap between patient requirements and the practice and experience of healthcare professionals. A more systematic evaluation of pain must be encouraged, particularly for outpatients, and with the use of adapted tools. The specific scales were revised to evaluate pain in children with disabilities and with a multidimensional approach to pain assessment and management, including physiologic, sensory affective, cognitive, behavioral, and sociocultural dimensions of pain. More courses about pain at medical universities should be encouraged, as at some universities, students spend only a few hours studying pain management. Caregiver training is needed, and close collaboration with pain centers, such as coconsultation with all the specialists at the same time, is encouraged. Finally, detailed pain detection and management protocols are recommended for these patients to improve our current practices. Since pain is an essential part of quality of life, it seems imperative to develop a routine pain assessment protocol for MPS patients that covers the entire spectrum of pain and that will be adapted for every type of patient, including those with neurocognitive and motor impairments. This initiative should be the subject of future prospective work from a national perspective.

## Electronic supplementary material

Below is the link to the electronic supplementary material.

Supplementary Material 1

## Data Availability

The datasets used and/or analyzed during the current study are available from the corresponding author upon reasonable request.
