# Supplementary material for: Pain assessment and treatment in patients with mucopolysaccharidoses: a French multicentric pediatric study
Source: Orphanet J Rare Dis. 2025 Nov 12;20:581. doi: 10.1186/s13023-025-04065-9 (PMC12613516; doi:10.1186/s13023-025-04065-9)

**Table S1: Clinical symptoms of the 48 MPS patients**

| Clinical symptoms | n (%) |
| --- | --- |
| Skeletal and joint abnormalities | 36 (75%) |
| Ear, nose and throat (ENT) symptoms | 34 (71%) |
| Cardiologic symptoms | 25 (52%) |
| Psychomotor disability | 24 (50%) |
| Respiratory symptoms | 20 (42%) |
| Carpal tunnel syndrome (CTS) | 18 (38%) |
| Ophthalmologic disease | 15 (31%) |
| Epilepsy | 7 (15%) |

*The results are expressed as the number of patients (%).*

**Table S2: Equipment, supportive care, and analgesic drugs**

|  | n (%) |
| --- | --- |
| Equipment* | 42 (86%) |
| Supportive care | 46 (98%) |
| Physiotherapy | 39 (81%) |
| Speech therapy | 17 (35%) |
| Psychology | 16 (33%) |
| Occupational therapy | 13 (27%) |
| Psychomotricity | 12 (25%) |
| Rehabilitation center | 9 (19%) |
| Balneotherapy | 8 (17%) |
| Other** | 10 (21%) |
|  |  |
| Regular use of analgesic drugs | 14 (29%) |
| Nonopioid analgesics (acetaminophen, NSAIDs) | 13 (27%) |
| Weak opioid agents (tramadol, codeine) | 0 |
| Strong opioid agents (morphine) | 0 |
| Other analgesic drugs (gabapentin, amitriptyline, other) | 3 (6%) |

*The results are expressed as the* *number of patients (%). *Equipment includes wheelchairs, hearing aids, glasses, implantable chambers, orthopedic aids, and noninvasive ventilation. **Other supportive care includes equitherapy, relaxation, vestibular rehabilitation, osteopathy, and orthoptics.*

**
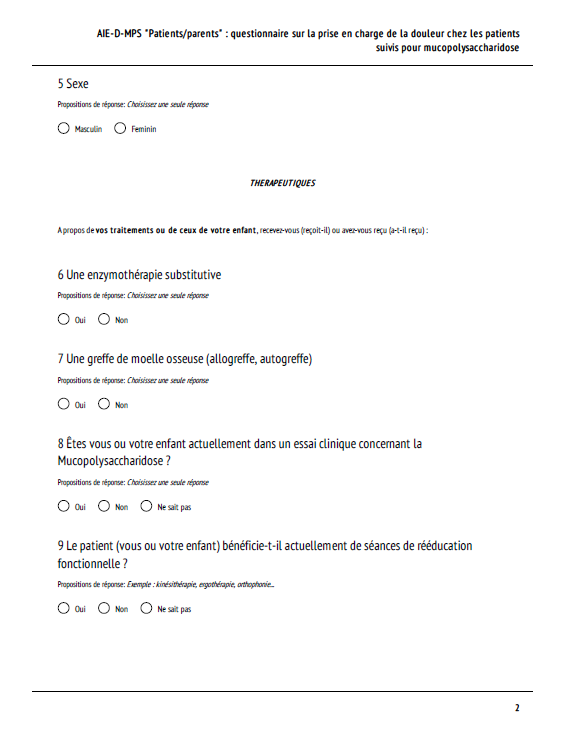
Online questionnaire for patients and/or parents**


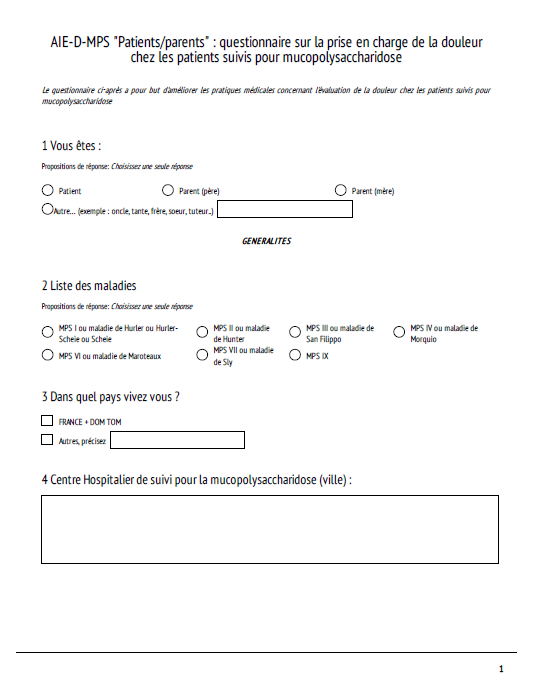


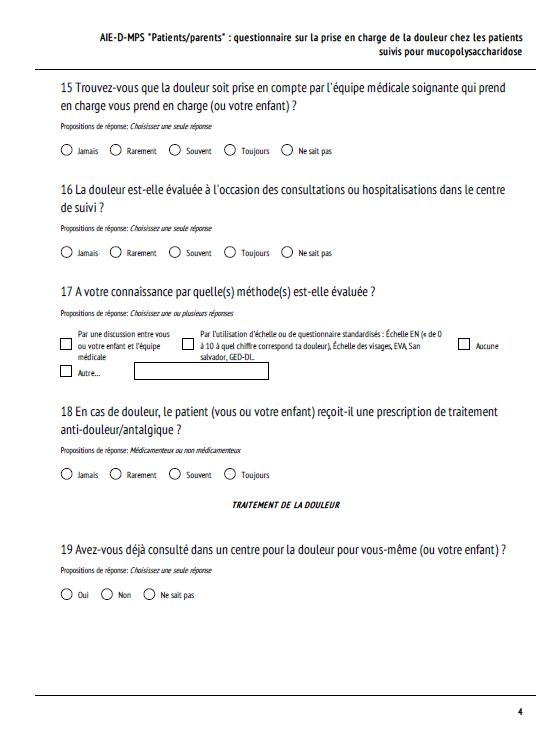

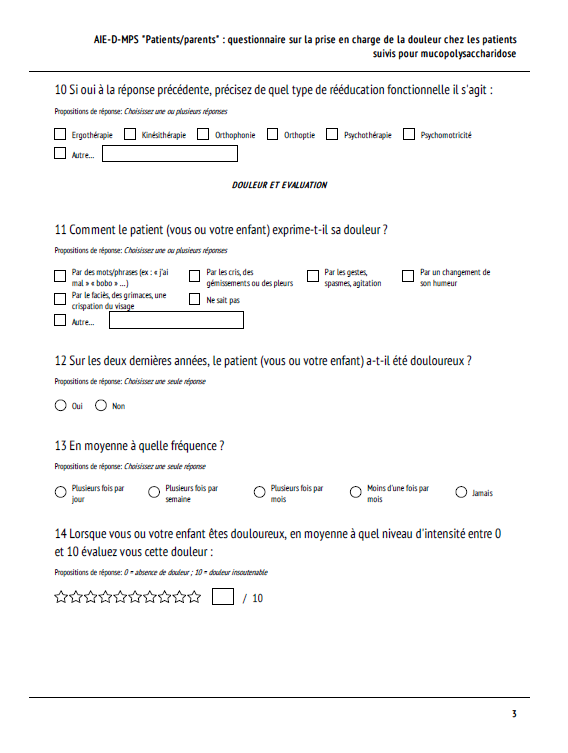


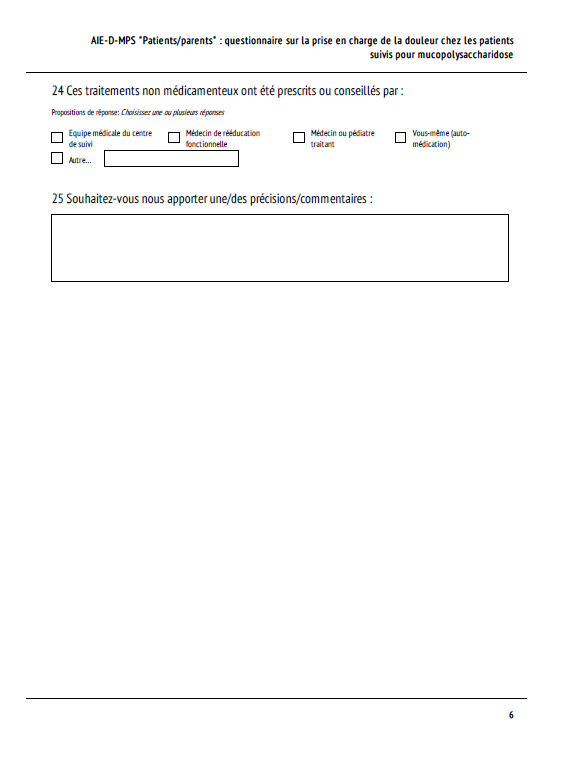

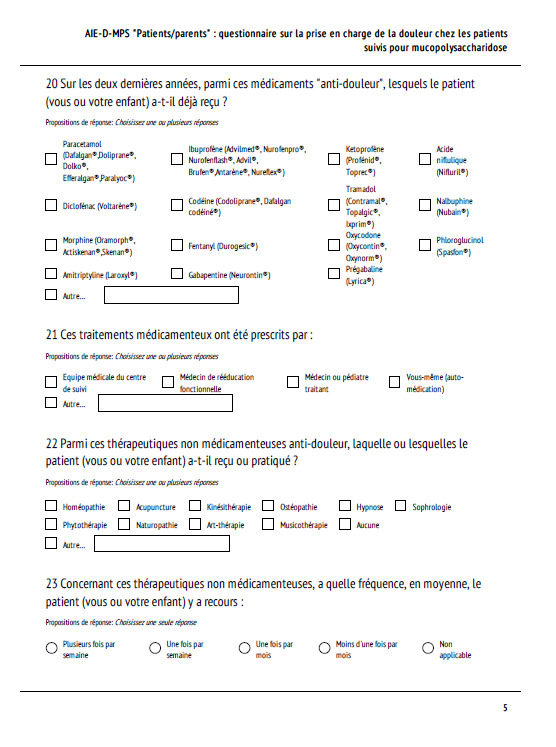


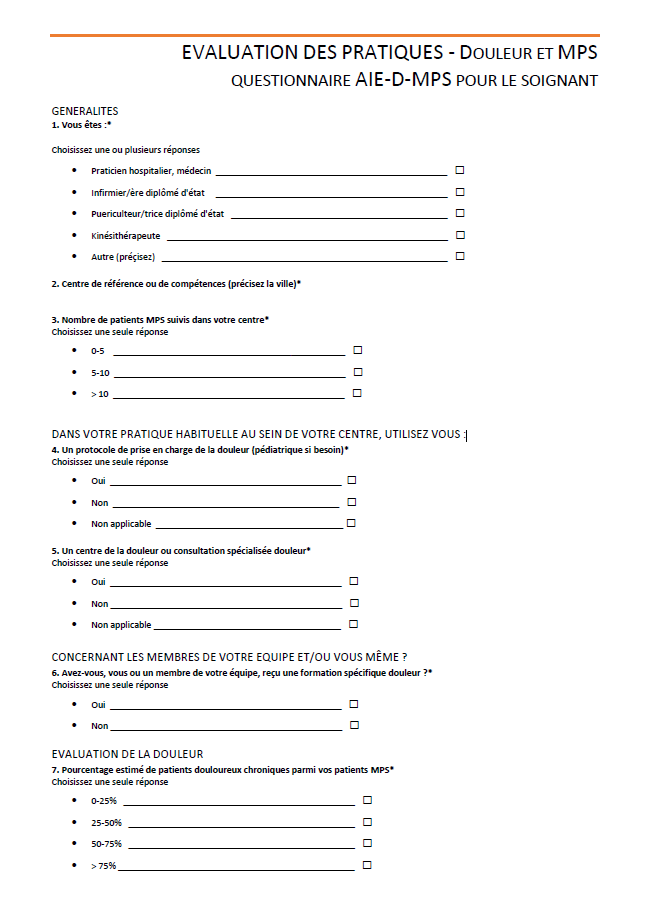
**Online questionnaire for healthcare professionals**


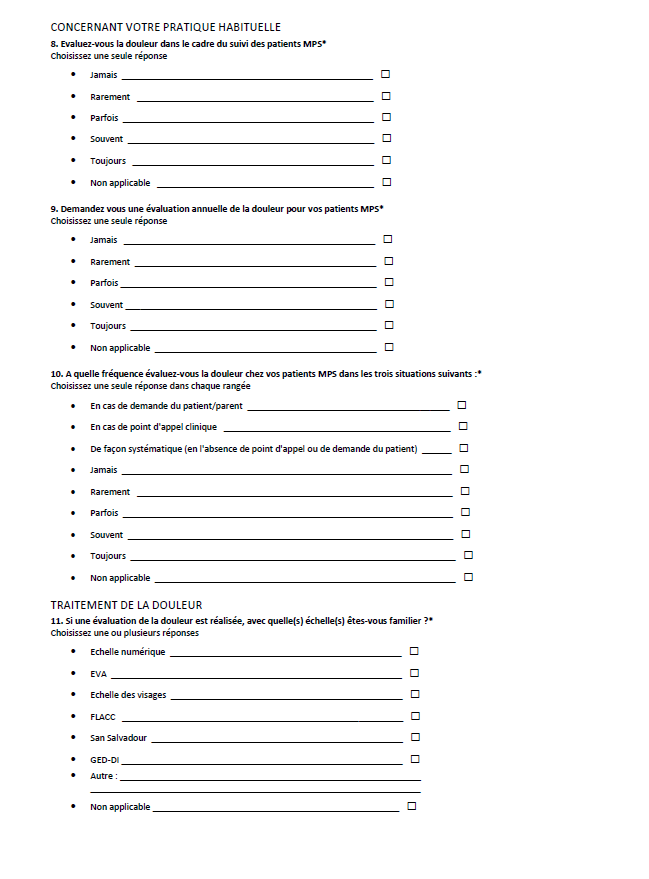


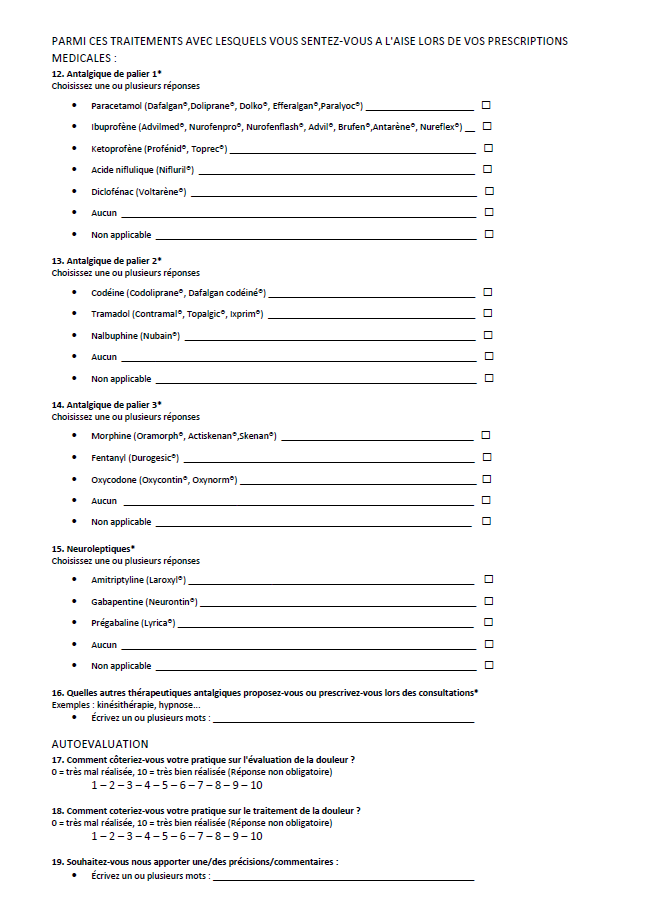

Supplement: Supplementary file 1 — Supplementary Material 1 [file 13023_2025_4065_MOESM1_ESM.docx]
